# What is known about neuroplacentology in fetal growth restriction and in preterm infants: A narrative review of literature

**DOI:** 10.3389/fendo.2022.936171

**Published:** 2022-08-19

**Authors:** Barbara Gardella, Mattia Dominoni, Annachiara Licia Scatigno, Stefania Cesari, Giacomo Fiandrino, Simona Orcesi, Arsenio Spinillo

**Affiliations:** ^1^ Department of Obstetrics and Gynecology, Fondazione Istituti di Ricovero e Cura a Carattere Scientifico (IRCCS) Policlinico San Matteo, Pavia, Italy; ^2^ Department of Obstetrics and Gynecology, University of Pavia, Pavia, Italy; ^3^ Department of Pathology, Fondazione Istituti di Ricovero e Cura a Carattere Scientifico (IRCCS) Policlinico San Matteo, Pavia, Italy; ^4^ Department of Brain and Behavioural Sciences, University of Pavia, Pavia, Italy; ^5^ Child Neurology and Psychiatry Unit, Istituti di Ricovero e Cura a Carattere Scientifico (IRCCS) Mondino Foundation, Pavia, Italy

**Keywords:** neuroplacentology, placental pathology and neurological outcome, neurological morbidity, placental epigenetic and neurodevelopment, cerebral palsy

## Abstract

The placenta plays a fundamental role during pregnancy for fetal growth and development. A suboptimal placental function may result in severe consequences during the infant’s first years of life. In recent years, a new field known as neuroplacentology has emerged and it focuses on the role of the placenta in fetal and neonatal brain development. Because of the limited data, our aim was to provide a narrative review of the most recent knowledge about the relation between placental lesions and fetal and newborn neurological development. Papers published online from 2000 until February 2022 were taken into consideration and particular attention was given to articles in which placental lesions were related to neonatal morbidity and short-term and long-term neurological outcome. Most research regarding the role of placental lesions in neurodevelopment has been conducted on fetal growth restriction and preterm infants. Principal neurological outcomes investigated were periventricular leukomalacia, intraventricular hemorrhages, neonatal encephalopathy and autism spectrum disorder. No consequences in motor development were found. All the considered studies agree about the crucial role played by placenta in fetal and neonatal neurological development and outcome. However, the causal mechanisms remain largely unknown. Knowledge on the pathophysiological mechanisms and on placenta-related risks for neurological problems may provide clues for early interventions aiming to improve neurological outcomes, especially among pediatricians and child psychiatrists.

## Introduction and aim of the study

The placenta represents the interface between the mother and the fetus; for this reason, the study of placental histological lesions and neonatal outcomes has spread greatly in recent years. As we all know, the placenta plays a fundamental role in the growth and development of the fetus, not only by allowing the transport of nutrients and oxygen and removing waste products from the fetal circulation (1), but also by constituting a protective and selective barrier for the passage of hormones, neurotransmitters, toxic agents and infectious microorganism (2, 3).

A compromised placental function may result in a higher risk of morbidity of the fetus and it may have consequences during the infant’s first years of life, but also later during adulthood.

Barker et al. firstly discovered the link between the nutritional environment *in utero* and cardiovascular morbidities in adults (4). From that moment, other adult chronic diseases, such as type 2 diabetes, insulin resistance, obesity, hypertension and cardiovascular disorders, have all been linked to fetal programming (5) in which the placenta plays a key role.

In recent years, a new field known as “neuroplacentology” has emerged: it focuses on the placenta’s role in protecting and shaping fetal and neonatal brain development.

As seen for other pathologies, placental failure can directly damage the developing brain or increase its susceptibility to injury, leading to possible permanent neurological disabilities (6). By adverse neurological outcomes, we observe organic lesions (periventricular leukomalacia, intraventricular hemorrhages, and strokes), alterations in neurocognitive development and neuropsychiatric disorders (autism spectrum disorders) and motor deficits which can arise both immediately after birth and in subsequent years (neonatal encephalopathy) (6–9).

However, the increasing literature data about neuroplacentology need a review in order to understand the relationship between placental histological lesions and impaired neurodevelopmental outcomes. In our opinion, it is very important to highlight the fact that placental unit dysfunction impairs the neurological development in two categories of newborn: fetal growth restriction and preterm.

## Materials and methods

We consulted the most significant medical databases, including PubMed, Cochrane Database of Systematic Reviews, EMBASE, and Web of Science, according to a combination of the following keywords: neuroplacentology, placental pathology and neurological outcome, neurological morbidity, placental epigenetics and neurodevelopment, and cerebral palsy, including pluralization and US English/UK English spelling variations and suffixes/prefixes.

For our analysis, all papers published online from 2000 until February 2022, including literature reviews, case series, and retrospective or prospective trials, were considered. We performed the research by employing a narrative review method (10, 11).

The first selection was based on the title, the second on the abstract, and the third on the full-text article.

We selected for analysis, study in which placental lesions were related to neonatal morbidity and short-term and long-term neurological outcomes. Therefore, we included only papers in which the following criteria were met: (I) description of histological placental lesions linked to adverse neurological outcomes (II) types of neurological injuries induced by placental impairment (III) most frequent placental functional modifications in newborns with a major risk of developing neurological disease, (IV) Redline classification of placental lesions (12).

The selected articles were assessed as full-text and resulting information was tabulated.

Exclusion criteria were as follows: (I) case reports considered to be of minor significance in this field were excluded by the present literature review; (II) abstracts of medical conferences, editorials, and preliminary studies; (III) multimedia materials regarding the aim of the study; (IV) papers written in languages other than English, (V) studies regarding the TORCH complex, (VI), studies regarding twin pregnancy, and chromosomal abnormalities.

Bias across studies as well as bias and risks related to the source of funding and conflict of interest of authors of the included studies, were assessed. Eventual disagreements were resolved through discussion.

## Results

For our purpose, a total of 1500 articles were identified by a searching strategy and 10 were identified through the references. Duplicated papers, presented in more than one database, and irrelevant works were not considered for our analysis; furthermore, after removing articles not published in English and published before 2000, 500 articles were screened by title and/or abstract. Full-text articles were assessed on the remaining 80 studies.

Finally, 27 studies were included and compared (**Table 1**). Among these, 6 studies focused on long term neurological outcomes (14, 20, 24, 25, 31, 40) with follow-up periods between 2 weeks (40) and 8 years (14).

**Table 1:** Studies included in the review.

	Reference	Number of patients	Results and conclusions
Acute placental dysfunction			
	Redline et al. (2005) (13)	83 term babies with NE Vs control group	Acute severe fetal placental vascular lesions are correlated highly with NI and CP and were found in 51% of index cases versus 10% of the comparison group (P<.0001). Prevalence of these lesions in the 64 infants with CP was 52% (P <.0001)
Maternal vascular malperfusion			
	Redline et al. (2007) (14)	129 ELBW infants who entered into a long-term follow-up program (8 years of age)	Placental lesions associated with maternal vascular underperfusion were risk factors for CP, while villous edema was associated with low scores on neurocognitive tests
	Straughten et al. (2017) (15)	55 ASD term babies VS 199 controls	MVM pathology (OR = 12.29; 95% CI = 1.37, 110.69) was associated with an increased risk of ASD. Acute placental inflammation was associated with an increased risk of ASD (odds ratio [OR] = 3.14, 95% CI = 1.39, 6.95).
	Ueda et al. (2022) (16)	258 infants with a follow up at 10, 14, 18, 24, 32, and 40 months	MVM is associated with the progression of infantile neurodevelopment during 10–40 months of age.
Fetal vascular malperfusion			
	McDonald et al. (2004) (17)	816 term newbors vs 387 controls Placental data were available for 93 cases (which were the final cohort)	The association between FTV placental findings and NE is statistically significant, with a P <0.05
	Vik et al. (2018) (18)	73 term babies with neonatal encephalopathy VS 253 controls	FVM of subacute or chronic origin was associated with increased risk of neonatal encephalopathy: global FVM were more frequent in case (20%) than control (7%) placentas (P = .001).
	Geraldo et al (2020) (19)	5 babies (2 preterm and 3 at term) with perinatal arterial ischemic stroke	The most plausible mechanism that links FVM to brain injuries is a thromboembolic phenomenon. High-grade FVM is associated with a higher risk of brain injury.
	Gardella et al. (2021) (20)	249 FRG and preterm babies of which 198 undergone 2-year follow-up	In preterm IUGR, FVM is correlated with an increased risk of abnormal infant neurodevelopmental outcomes at 2 years of age even in absence of neurological abnormalities at discharge from the NICU. The rate of major and minor neurodevelopmental sequelae was 57.1% (4 of 7) among severe FVM (adjusted odds ratio, 24.5; 95% confidence interval, 4.1e146).
Chorioamnionitis			
	Redline et al. (2000) (21)	40 term infants with neurologic impairment VS 176 consecutive meconium-stained term infants	Severe fetal CA is independently associated with NI (odds ratio [OR], 13.2; 95% CI, 1.2–144); and the risk of NI increased as a function of the number of lesions present (OR, 10.1; 95% CI, 5.1–20 for each additional lesion).
	Polam et al. (2005) (22)	177 VLBW babies	Infants with CA, compared with controls, had a significantly higher incidence of IVH (30% vs 13%) and ROP (68% vs 42%).
	Wintermark et al. (2010) (23)	23 asphyxiated newborns at term	CA with fetal vasculitis and chorionic plate meconium were significantly associated with brain injury (P=0.03).Therapeutic hypothermia may not be effective in asphyxiated newborns whose placentas show evidence of chorioamnionitis with fetal vasculitis and chorionic plate meconium
	Rovira et al. (2011) (24)	177 preterm infants undergone 2-year follow up	Infants with funisitis, compared with controls, had a significantly higher incidence of moderate to severe disability (18% vs 5%, OR 4.07; 95% CI 1.10–15.09).
	Van Vliet et al. (2012) (25)	51 very preterm infants followed up at 2 and 7 years of age	At 2 years, very preterm infants with placental underperfusion had poorer mental development than very preterm infants with HCA (mean [SD] 90.8 [18.3] vs 104.1 [17.2], adjustedd =1.12, P = .001). Motor development was not different between both groups (92.8 [17.2] vs 96.8 [8.7], adjusted d = 0.52, P=0.12).
	Hayes et al. (2013) (26)	141 term newborns with neonatal encephalopathy VS 309 controls	Meconium phagocytosis, haemorrhage, and/or markers of infection/inflammation were independently associated with NE (p <0.05) and showed a synergistic effect, when combined, for short- and long-term impairments.
	Lachapelle et al. (2015) (27)	142 asphyxiated newborns at term	Among the asphyxiated newborns, the placental microscopic findings tended to be more common in those developing brain injury compared to those who did not: chorionic plate meconium in 50% compared to 36%, CA in 75% compared to 44%, and villitis of unknown etiology in 67% compared to 33%
	Mir et al. (2015) (28)	120 term babies with neonatal encephalopathy	CA with or without fetal response, and patchy/diffuse chronic villitis were found to be independently associated with severity of NE (P <0.001).
	Anblagan et al. (2016) (29)	90 preterm infants	Diffuse white matter injury begins in utero for a significant proportion of preterm infants and HCA is a risk factor (p<0.05)
	Raghavan et al. (2019) (30)	1031 term (49.6%) and preterm babies (50.4%)	PTB was an independent risk factor for NDDs. Placental HCA (CA) and PTB additively increased the odds of NDDs (aOR: 2.16, 95% CI: 1.37, 3.39), as well as ADHD (aOR: 2.75, 95% CI: 1.55, 4.90), other developmental disabilities (aOR: 1.96, 95% CI: 1.18, 3.25) and possibly ASD (aOR: 2.31, 95% CI: 0.99, 5.39).
Epigenetic modifications			
	Elbers et al. (2011) (31)	12 cases of neonatal stroke born at term	Multiple risk factors are involved in neonatal stroke, and placental pathology may be a contributing factor.
	Harteman et al. (2013) (32)	95 full-term infants with neonatal encephalopathy	Decreased placental maturation and hypoglycemia <2.0 mmol/L were associated with increased risk of white matter/watershed injury with or without basal ganglia and thalamic involvement (OR, 5.4; 95% CI, 1.4-21.4). Chronic villitis was associated with basal ganglia and thalami injury irrespective of white matter injury (OR, 12.7; 95% CI, 2.4-68.7).
	Roescher et al. (2014) (33)	52 preterm infants who undergone 2 weeks follow up	Placental lesions were not associated with infants’ neurological motor development during the first two weeks after birth in preterm infants
	Paquette et al. (2015) (34)	537 term babies	methylation patterning of glucocorticoid response genes influences neurobehavior
	Schmidt et al. (2016) (35)	47 placentas of children clinically diagnosed at 3 years with ASD	Abnormal Placental DNA methylation is a possible mechanism for ASD. The strongest, most robust associations were between pesticides professionally applied outside the home and higher average methylation over PMDs [0.45 (95% CI 0.17, 0.72), P¼0.03].
	Chang et al. (2017) (36)	89 high ASD risk newborns at term VS 201 cont(rols	Placental chorionic surface vascular network associated with placentas of high-risk ASD pregnancies generally had fewer branch points, thicker and less tortuous arteries, better extension to the surface boundary, and smaller branch angles than their population-based counterparts
	Wu et al. (2017) (37)	Mice model	IL-6 activation in placenta is required for relaying inflammatory signals to the fetal brain and impacting behaviors and neuropathologies relevant to neurodevelopmental disease.
	Park et al. (2018) (38)	129 high ASD risk newborns at term VS 267 controls	Findings suggest that there may be some gross morphological differences between general population and high ASD risk placentas: the placentas of ASD-case siblings were rounder and more regular in perimeter than general population placentas (p<0.05). No significant differences were observed in cord insertion measures.
	Vacher et al. (2021) (39)	Mouse model	Abnormal placental endocrine function is linked to diverse neurodevelopmental disorders, cerebellar development and social behavior, in particular a reduction of ALLO alters neurodevelopment in a sex-linked manner

ADHD, attention-deficit/hyperactivity disorder; HCA histological chorioamnionitis, ALLO, allopregnenolone; IUGR, intrauterine growth restriction; ASD, autism spectrum disorder; MVM, maternal vascular malperfusion; CA, chorioamnionitis; NDD, neurodevelopmental disorders; CI, confidence interval; NE, neonatl encephalopathy; CP cerebral palsy; NI, neurological impairment; ELBW, extremely low birth weight; NICU neonatal intensive care unit; FTV fetal thrombotic vasculopathy; ROP retinopathy of premature; FVM fetal vascular malperfusion.

### Acute placental dysfunction

Acute placental dysfunction regards the main placental lesions found in term newborns affected by neurological impairments. The correlation between neurological injuries and placenta pathology was investigated in term newborns in 9 of the research analyses included in the review (13, 17, 21, 23, 26–28, 34).

The main placental lesions investigated were acute interruption of placental circulation, such as in *abruptio placentae* or umbilical cord true knots, thrombosis, wrapping and torsion. These conditions commonly correlate with neurological hypoxic-ischemic injuries both before and/or during delivery (13, 23, 27, 41, 42) which are neonatal encephalopathy (13, 18, 21), neonatal stroke (31, 43), periventricular leukomalacia and intraventricular hemorrhage (13, 18, 21).

### Maternal vascular malperfusion and fetal vascular malperfusion

On the other hand, most of the literature research has focused on preterm and FGR newborns because of their close connection with neurological injuries. In these fetuses the most frequent vascular placental lesions are maternal vascular malperfusion and fetal vascular malperfusion (13, 44).

Maternal vascular malperfusion (MVM) is predominantly associated with an increased risk of autism spectrum disorders 15, but it is also correlated with cerebral palsy, periventricular hemorrhage (14, 25).

In several research analysis, fetal vascular malperfusion (FVM) has been linked to neonatal encephalopathy and cerebral palsy, particularly in term infants (13, 17, 18), while in preterm babies it may be responsible for lower 2-year neurodevelopmental general quotient (GQ) by Griffiths’ Scales, in particular referring to personal-social abilities, hearing, speech and performance subscales scores (20), while it seems to not be associated with short-term neonatal outcomes (20).

### Chorioamnionitis

Histological chorioamnionitis has been linked to the etiology of neuropsychiatric disorders, including generalized cognitive impairment, autism spectrum disease and schizophrenia (45–50). However, white matter brain lesions (51–53) and abnormalities in social behavior, complex learning tasks and sensorimotor gating (46–48) represent the most significant adverse outcomes related to chorioamnionitis. Furthermore, if associated with funisitis and fetal thrombotic vasculopathy, chorioamnionitis is involved in the development of neonatal encephalopathy (21, 23, 26, 28), and of intraventricular hemorrhage (22, 51, 54–56).

On the other hand, chronic villitis is also correlated with neonatal encephalopathy, nevertheless it is a non-infectious inflammatory process (17, 26, 28, 32).

### Abnormal placental morphology

A different placental morphology may be associated with some altered neurological outcomes. It was reported that the placentas of fetuses affected by autism spectrum disorder (ASD) appeared thicker and rounder (38). Instead, Chang et al. observed some anatomical variations in the placentas of a cohort with an elevated risk of ASD, in particular in the vascular architecture: the placental arteries were thicker and less tortuous, more extended to the surface but with fewer branches and smaller branch angles than population-based counterparts (36). The placentas analyzed in this study (36) were taken from two independently collected cohorts, Early Autism Risk Longitudinal Investigation (EARLI) and National Children’s Study (NCS): EARLI includes pregnancies for a high risk of autism because it focuses on the prenatal and early life periods of children who have biological siblings affected by ASD (57). NCS is a cohort of pregnancies with an unknown risk for ASD, where placentas were used by Chang as an ASD low-risk population.

### Epigenetic modifications

Nowadays, proteomic and metabolomic studies regarding placental dysfunction have found abnormal synthesis of glucocorticoids, due to maternal stressors (such as malnutrition or hypoxia) or barrier dysfunction. The exposition of fetal tissues to high levels of glucocorticoids (in particular cortisol), may lead to epigenetic changes (which are altered DNA methylation and altered mRNA expression) (58, 59) and may disturb the trajectory of multiple neurodevelopmental processes (60).

In 2015, Paquette et al. have demonstrated that methylation patterning of glucocorticoid response genes influences neurobehavior through quantification of placental methylation using bisulfite pyrosequencing in 537 term infant placentas and analyzing profiles of neurobehavior *via* the Neonatal Intensive Care Unit Network Neurobehavioral Scales (46).

Furthermore, the placenta is responsible for the production of neurotransmitters, such as serotonin, dopamine, norepinephrine and epinephrine: anything that compromises their passage from the placenta to the the fetal brain can increase the risk for neurobehavioral disorders (61).

## Discussion

The possible mechanisms through which placenta can impact brain development are different. It is thought that an antenatal and/or an intra-partum one (6, 14, 17, 21), as well as both acute and chronic placental dysfunctions (41) are responsible for possible neurological impairments. The impact of such events depends on when they occur during gestation (41) and the histopathological examination of the placenta at the time of delivery is useful to understand the timing of the exposure during pregnancy (62).

For this reason and to highlight the importance of placental abnormal mechanisms and how they could impact neurodevelopment, we regrouped the articles in different sections: acute placental dysfunction, chorioamnionitis, MVF, FVF, and epigenetic and metabolic placental alterations (**Table 1**). Furthermore, for each section, we distinguished between term and preterm and/or FRG newborns.

Because the heterogeneity of placental lesions associated with adverse neurological outcomes reflects different pathways, we have listed the works in chronological order with the aim of not being a fully exhaustive review but only of highlighting the main points for the reader’s knowledge.

The commonalities and the correlations between placental lesions and fetal neurological outcomes are shown in **Table 2**.

**Table 2:** Correlations between selected placental lesions and fetal neurological outcomes.

	*Acute placental dysfunction*	*Maternal vascular malperfusion (MVM)*	*Fetal vascular malperfusion (FVM)*	*Epigenetic modifications*	*Chorionamnionitis*
**Neonatal encephalopathy**	**✓**		**✓**		**✓**
**Neonatal stroke**	**✓**				
**Periventricular leukomalacia**	**✓**				**✓**
**Intraventricular hemorrhage**	**✓**				**✓**
**Autism spectrum disorders**		**✓**		**✓**	**✓**
**Cerebral palsy**		**✓**	**✓**		
**Periventricular hemorrhage**		**✓**			
**Cognitive impairment**			**✓**		**✓**
**Neuropsychiatric disorders**					**✓**
**Neurobehavioral disorders**				**✓**	

### Acute placental dysfunctions

Predominantly, they include the acute interruption of placental circulation, such as in *abruptio placentae* or umbilical cord true knots, thrombosis, wrapping and torsion, and commonly correlate with neurological hypoxic-ischemic injuries both before and/or during the delivery (13, 23, 27, 41, 42).

The complex causal pathway underlying how these placental injuries could bring to neurodevelopmental impairment is poorly understood, probably because only recently attention was paid to the lesions that affect the fetal placental side including fetal thrombosis, inflammation of the fetal vessel wall, and hemodynamically significant umbilical cord abnormalities. The fetal placental bed receives up to 55% of the total fetal cardiac output. Probably, these lesions influence fetal neurodevelopment through different pathways: an impaired fetoplacental vascular regulation, a decreased gas and metabolite exchange, the activation of platelets and leukocytes, the generation of cytokines and other thromboinflammatory mediators, the release of heat shock proteins from ischemic placental tissue, and embolism of placental thrombi to other fetal vascular beds (13).

### Chronic placental dysfunction

However, most research regarding the role of placental lesions in neurological development has been conducted on IUGR and preterm infants, because these conditions are strongly associated with pathology of the placenta (63) and because prematurity is an independent risk factor for neurodevelopmental disabilities (30).

Histological chorioamnionitis, maternal vascular malperfusion, fetal vascular malperfusion and fetal thrombotic vasculopathy are the most common placental injuries associated with adverse neurological outcomes in preterm and FGR births (8, 20, 24, 25, 30, 64–66).

### Fetal vascular malperfusion

Fetal vascular malperfusion, a term introduced by the Amsterdam International Consensus group of placental pathologists in 2015, indicates a reduced or absent perfusion of the villous parenchyma by the fetus (67). The most common etiology of FVM is umbilical cord obstruction, while other possible contributing factors are maternal diabetes, fetal cardiac insufficiency or hyperviscosity, inherited or acquired thrombophilias (67). Both fetal coagulopathy or a maternal hypercoagulable state, antiphospholipid antibody syndrome or antiplatelet antibodies which increase the likelihood of thrombosis (68).

Regarding brain injuries, fetal vascular malperfusion (FVM) has been associated with neonatal encephalopathy, cerebral palsy (13, 17, 18), and lower personal-social abilities, hearing, speech and performance subscales scores (20).

The most plausible mechanism that links FVM to brain injuries is a thromboembolic phenomenon. Indeed, it is believed that FVM-related thromboemboli spread into the venous fetal circulation, reaching the cerebral circulation through the right atrium, the foramen ovale and the fetal ductus arteriosus (69, 70). Vascular occlusion could interest both arterial and venous systems, even simultaneously (71).

Some common risk factors underlying FVM, activating coagulation and inflammatory pathways in both the arterial and venous system or a secondary reduction of venous flow in the dural sinuses, can be contributing factors (71, 72).

High-grade FVM is associated with a higher risk of brain injury (73), as demonstrated by Geraldo et al. in their case series: they described the clinical-neuroimaging features of 5 neonates with arterial ischemic stroke and highlighted that all the patients were classified as having high-grade FVM (19).

### Maternal vascular malperfusion

Maternal vascular malperfusion (MVM) consists of a group of placental gross and histological findings regarding both maternal decidual vessels, reflecting abnormal spiral artery remodeling, and in the villous parenchyma, reflecting abnormalities in oxygenation and flow dynamics in the intervillous space (74). It is common in pregnancies complicated by preeclampsia and FGR, oligohydramnios, and stillbirth (75). The exact etiopathological mechanism that leads to MVM is not clear, but it seems that a defective deep spiral artery remodeling at the junctional zone is a fundamental process (74). It could be caused by a hypoxic-ischemic injury which leads to oxidative stress of the intervillous space (76).

Severe MVM is often associated with autism spectrum disorders (15), cerebral palsy, periventricular hemorrhage (14, 25). Probably, MVM may be responsible for hypoxic conditions in fetal blood, which may contribute to altered neurodevelopment during the early infantile period (16).

### Chorioamnionitis

Chorioamnionitis is the inflammatory involvement of the chorion and the amnios, while we talk about funisitis, if the inflammation affects the umbilical cord, and of villitis when it affects the villous tree (77). From a histopathological point of view, they are all characterized by the infiltration of neutrophils (77). One of their most frequent causes is an intra-amniotic infection which could reach the amniotic cavity from the lower genital tract (78), from the maternal blood (79, 80), during invasive procedures (81) and maybe from the peritoneal cavity through the fallopian tubes.

Ascending microbial infection is the most frequent mechanism for intra-amniotic infection (81) and the most frequent microorganisms found are Ureaplasma, Gardnerella vaginalis, Fusobacterium species, Candida albicans (81). Rupture of membranes is not necessary for bacteria to reach the amniotic cavity (82).

But chorioamnionitis is also caused by a “sterile inflammation” (83) and possible mechanisms are: inflammatory processes as non-specific mechanisms of host defense against danger signals of non-microbial origin, extra-amniotic infection, non-viable microorganisms which may release chemotactic factors leading to inflammation (81).

Several studies have demonstrated the link between histological chorioamnionitis and various forms of neurodevelopmental impairment such as white matter brain lesions (51–53) and abnormalities in social behavior, complex learning tasks and sensorimotor gating (46–48), neuropsychiatric disorders (45–50), neonatal encephalopathy (21, 23, 26, 28), and intraventricular hemorrhages (22, 51, 55, 56).

It is believed that chorioamnionitis, leading to the activation of the maternal immune system, could trigger a fetal inflammatory response (FIRS) with a release of proinflammatory cytokines (84) and local thrombosis in severely inflamed vessels (85) that could directly impact the immature brain and increase its susceptibility to neurodevelopmental disorders (13, 51, 86–88).

### Epigenetic modifications

Finally, placental epigenetic changes are linked to altered neurodevelopment. From this point of view, these modifications consist predominantly in altered DNA methylation and altered mRNA expression (58, 59) and are influenced by clinical pregnancy features and environmental exposure to toxins (60).

These modifications can be caused by maternal stressors, such as hypoxia or malnutrition, which, for example, could impact on the synthesis of glucocorticoids in the placenta (89, 90). Maternal obesity increases the risk of long-term neurological impairment and psychiatric disorders (91–93).

As described by Cirulli, exposure to a poor socioeconomic environment characterized by stress, maternal depression and/or maternal obesity can lead to increased risk for neuropsychiatric diseases, cognitive impairment and Alzheimer’s disease (94)

Confirming this, MARBLES study suggested that pesticide exposure could alter placental DNA methylation more than other factors (35). Also, vitamin D deficiency (DVD) can contribute to placental cytokine response and neurobehavioral outcomes: indeed, higher concentrations of 25(OH)D during pregnancy were associated with a decreased probability of autistic phenotypes (95).

Glucocorticoids (GCs) and neurotrophins have important effects on brain plasticity (94).

In perspective, another key placental hormone in shaping the fetal brain might be placenta allopregnanolone (ALLO), a major GABAergic neurosteroid, synthesized from progesterone. It was demonstrated that ALLO is a potent regulator in many neurodevelopmental processes, including neurogenesis, neuritogenesis, cell survival, synapse stabilization and myelination (96) and the decrease of its levels leads to increased apoptosis, excitotoxicity and impaired myelination, particularly in males (97). Vacher et al. demonstrated that placental ALLO insufficiency led to cerebellar white matter abnormalities that correlated with autistic-like behavior only in male offspring (39) using a new conditional mouse model, in which the gene encoding ALLO’s synthetic enzyme (akr1c14) is specifically deleted in trophoblasts. A single injection of ALLO during late gestation abolished these alterations (39).

Nevertheless, the relation between placental lesions and short-term neurological injuries (like white matter diseases and intraventricular hemorrhage) in preterm infants has been widely studied, as we see also in this case the causal mechanism remains largely unknown.

Furthermore, Roescher et al. (33) reported that placental lesions were not associated with adverse neurological motor development during the first two weeks after birth in preterm infants.

On the other hand, it seems that placental pathology strongly correlates with long-term neurological outcomes of cognitive performance, particularly in cases of fetal vascular malperfusion (20). Regarding this, van Vliet et al. in 2012 (25) demonstrated that at 2 years, very preterm infants with placental underperfusion had poorer mental development than very preterm infants with histological chorioamnionitis, while no differences between either group were seen regarding motor development. This hypothesis has been confirmed by Gardella et al. (20). The authors reported that fetal vascular malperfusion is correlated with an increased risk of abnormal infant neurodevelopmental outcomes at 2 years of age even in the absence of brain lesions or neurological abnormalities at discharge (20).

However, placental dysfunction alone almost never is sufficient. Indeed, recent studies support the multifactorial pathogenesis hypothesis and the co-occurence of several risk factors being associated with many neurodevelopmental disorders (98, 99). Among these, preterm birth (especially if spontaneous and not medically indicated) (15, 52, 100–103, 29) and fetal growth restriction (14) are the principal ones, with all their respective risk factors (20, 104, 30, 105, 106). There is, however, also a maternal infectious or maternal inflammatory status involved (15, 107–111): indeed, maternal inflammation and cytokine production, especially interleukin-6, -2, and -17a (IL-6, IL-2, and IL-17a), are strongly linked to neurodevelopmental impairment in offspring (37, 112–114).

Regarding the limitations of our review: first of all this paper is a narrative review and not a systematic analysis due to heterogeneity of the considered placental lesions. In addition, the previous literature data derived from retrospective analyses in which the observational time of neurological development in newborns is different: only in 5 of the selected studies patients underwent at least a 2-year follow-up.

Finally, most studies included in this review were conducted in high-risk populations, such as IUGR and preterm infants. Studies in a low- or moderate risk group, such as term infants, in which the incidence of cerebral palsy is low, may reveal different results.

## Conclusion

The histological examination of placental tissue may be useful in particular in the follow-up of preterm and FGR newborns, because, even in the absence of neurological impairment at discharge, the likelihood of intact 2 year survival is lower, especially in those whose placenta presents FVM lesions.

Furthermore, in addition to histopathological analysis, proteomic and epigenetic evaluations of placenta may be fundamental for assessing the impact of injury in neurodevelopment.

We are conscious that more research is necessary regarding the pathophysiological mechanisms leading from placental injury to adverse neurological outcomes, with the aim of identifying other possible intrauterine risk factors and diagnostic biomarkers. This may help to identify a group of pregnancies and neonates at major risk of adverse neonatal outcomes with the aim of monitoring these infants more closely.

Especially interesting is the therapeutic aspect that such knowledge can influence for example the most appropriate use of therapeutic hypothermia in case of acute perinatal asphyxia and neonatal encephalopathy or developing hormone replacement strategies to maintain the normal neurodevelopment and protect the brain from further injury.

## Author contributions

ALS and MD wrote the manuscript. BG and AS designed the structure of the manuscript. AS, BG, SC, SO and GF contributed to the literature search. MD, BG, and AS reviewed and revised the initial manuscript and approved the final manuscript as submitted. All authors contributed to the article and approved the submitted version.

## Funding

The study is supported by grants of the Italian ministry of health, “ricercar corrente” to the IRCCS Fondazione Policlinico San Matteo, Pavia, Italy.

## Conflict of interest

The authors declare that the research was conducted in the absence of any commercial or financial relationships that could be construed as a potential conflict of interest.

## Publisher’s note

All claims expressed in this article are solely those of the authors and do not necessarily represent those of their affiliated organizations, or those of the publisher, the editors and the reviewers. Any product that may be evaluated in this article, or claim that may be made by its manufacturer, is not guaranteed or endorsed by the publisher.
